# The requirement for methodologically well-designed research in dentistry

**DOI:** 10.21142/2523-2754-1301-2025-225

**Published:** 2025-03-03

**Authors:** Luis Ernesto Arriola Guillén

**Affiliations:** 1 Division of Orthodontics, School of Dentistry, Universidad Científica del Sur. Lima, Peru. luchoarriola@gmail.com Universidad Científica del Sur Division of Orthodontics School of Dentistry Universidad Científica del Sur Lima Peru luchoarriola@gmail.com

Evidence-based dentistry relies on rigorous and structured research conducted by scientists leading well-designed studies. ^(^^1^ These studies must use representative samples taken from the populations they aim to assess. The quality and representativeness of a sample depend on two key factors: its size and the method used for sampling. ^(^^2^ In the materials and methods section of a research article, it is essential to provide a clear and comprehensive explanation of the formula used to calculate the sample size.^1^^,^^2^ Researchers must choose an appropriate sample size formula that aligns with the nature of the outcome variable and the number of groups they intend to compare.^2^ Unfortunately, a significant number of research articles fall short in this important area, often due to incomplete descriptions or the application of inappropriate formulas that can compromise the external validity of their findings.

Sampling techniques are essential for ensuring the reliability of research outcomes. Probabilistic sampling is considered the gold standard, as it ensures that every study unit has an equal opportunity to be selected for participation. ^(^^1^^,^^2^ A successful probabilistic sampling process requires a complete sampling frame, which is a comprehensive list of all potential study units. However, flaws in two key areas, "determining sample size and selecting an appropriate sampling technique," are often found in published scientific literature. This situation is particularly concerning, as the primary aim of publishing should be to produce high-quality articles on specific topics rather than succumbing to the pressures of rapid publication.

Another significant factor in the research process is selecting the most suitable study design tailored to the research question. ^(^^1^^,^^3^ In this way, many systematic reviews concluded the importance of employing well-structured study designs to ensure greater external validity, which enables the generalization of results to broader populations with similar selection criteria. ^(^^3^ Clinical trials in dentistry are relatively scarce, particularly in specialties where available studies are limited. Observational studies offer a viable alternative, yet they must meticulously control covariates to prevent any potential bias. Such studies should also incorporate a robust statistical analysis plan featuring appropriate multivariate analyses, which simultaneously account for all relevant covariates. ^(^^4^ This comprehensive approach allows researchers to assess better the significance of each variable's impact on the outcome variable. Unfortunately, this level of detailed analysis is rarely seen in published articles, which further diminishes their external validity.

Finally, research must detail the specific training and calibration procedures used for measuring variables, as these steps are essential for ensuring the validity and reliability of the reported results. ^(^^1^ Researchers should evaluate these procedures using recognized statistical tests, commonly the Kappa test for qualitative variables and the intraclass correlation coefficient (ICC) test for quantitative variables. ^(^^5^^,^^6^ Moreover, transparency is vital; thus, the outcomes of these tests and their confidence intervals should be clearly documented in the research to foster trust in the methodologies employed.

In summary, dental researchers across all specialties must consider the implementation of these rigorous standards and practices. By doing so, they can enhance the methodological integrity of their studies and ultimately produce results that have a more profound and positive impact on society.
